# Traditional Knowledge of Medicinal Plants Used in the Northeastern Part of Morocco

**DOI:** 10.1155/2021/6002949

**Published:** 2021-08-06

**Authors:** Mohamed Reda Kachmar, Hanae Naceiri Mrabti, Meryem Bellahmar, Abdelilah Ouahbi, Zoubida Haloui, Khalid El Badaoui, Abdelhakim Bouyahya, Said Chakir

**Affiliations:** ^1^Team of Medicinal and Aromatic Plants Valorisation and Environment, Environment and Health Laboratory, Faculty of Sciences, Moulay Ismail University, P. O. Box 11201, Zitoune, Meknes, Morocco; ^2^Laboratory of Pharmacology and Toxicology, Bio Pharmaceutical and Toxicological Analysis Research Team, Faculty of Medicine and Pharmacy, Mohammed V University, BP 6203, Rabat, Morocco; ^3^Laboratory of Human Pathologies Biology, Department of Biology, Faculty of Sciences, Genomic Center of Human Pathologies, Faculty of Medicine and Pharmacy, Mohammed V University, Rabat, Morocco

## Abstract

The knowledge of the plants that are used may provide insight on their properties for further exploration. This study aimed to identify and collect data about medicinal plants used in traditional medicine by the population of the provincial region of Taza, Morocco. An ethnobotanical survey was carried out among 200 informants, competent villagers, herbalists, and traditional healers from the provincial region of Taza city through direct interviews using a structured questionnaire. The survey reported 55 plant species belonging to 28 families used in the folk medicine. Informants' results showed that the most frequently used plants were *Origanum compactum*, *Mentha pulegium*, *Rosmarinus officinalis* L., *Aloysia citrodora*, *Calamintha officinalis* Moench, and *Artemisia herba-alba* Asso., with a relative frequency of citation of 76%, 72%, 60%, 42%, 40%, and 30%, respectively. Moreover, in this study, the Lamiaceae family was the most commonly reported plant family, and the leaves were the most frequently used parts of the plants; otherwise, decoction and infusion were the most used modes in the preparation of remedies from medicinal plants in the traditional medicine. The sociodemographic characteristics showed that women use medicinal plants slightly more than men, the illiterate people use the medicinal plant the most, and old people have more information about the medicinal plants than the new generations. The region of Taza of Morocco has an important floristic biodiversity of medicinal plants which are used in traditional medicine practice. This result provides a good database for pharmacological screening in the search for new plants that can contain new bioactive molecules that can be used as a bioactive ingredient of medicament or as a biological alternative in pharmacology.

## 1. Introduction

For a long time, plants have played a very important role in the daily lives of human life [1]. Herbal medicines have traditionally been used because of several benefits; they are affordable and easily accessible, and there is no evidence of resistance to whole plant extracts or of effectiveness [2]. This sort of traditional medicinal knowledge has been regularly practiced in homes and is transferred from generation to generation with the passage of time [3]. Nowadays, the use of plants as a way of treatment is still very important for many rural and urban Moroccans [4]. In recent decades, scientific studies have increasingly focused on plants used in traditional medicine to treat various diseases through botanical surveys and laboratory biological tests on animal models to discover certain species with medicinal properties that may replace certain chemical drugs with side effects [5, 6]. Morocco is a Mediterranean country which is crisscrossed from east to west and from southwest to northeast by four mountain ranges, the Rif, the Middle Atlas, the High Atlas, and the Anti-Atlas; his position between two seas and a vast desert results in a complete range of Mediterranean bioclimates. This varied climate provides habitat for rich and varied flora: more than 4200 spontaneous species and some 1500 introduced species have been catalogued [1, 7, 8]. The region of Taza is located between the mountain ranges of the Rif and the Middle Atlas; its climate is characterized by dry and very hot summer and cold, precipitating, and partly snowy winter. During the year, the temperature generally ranges from 5°C to 36°C and is rarely below 2°C or above 41°C. This climate makes this region very rich in plant biodiversity and in wide varieties of indigenous medicinal plants used by the local population in the folk medicine. To the best of our knowledge, few works in the literature were interested in this region. Thus, the aim of this work was to collect information about plant species used in folk medicine by the traditional healers and local population of this region to treat diseases and human pathologies. For these reasons, the current survey was conducted in the provincial area of Taza of Morocco (northeastern Morocco).

## 2. Materials and Methods

### 2.1. Study Area

**Figure 1:**
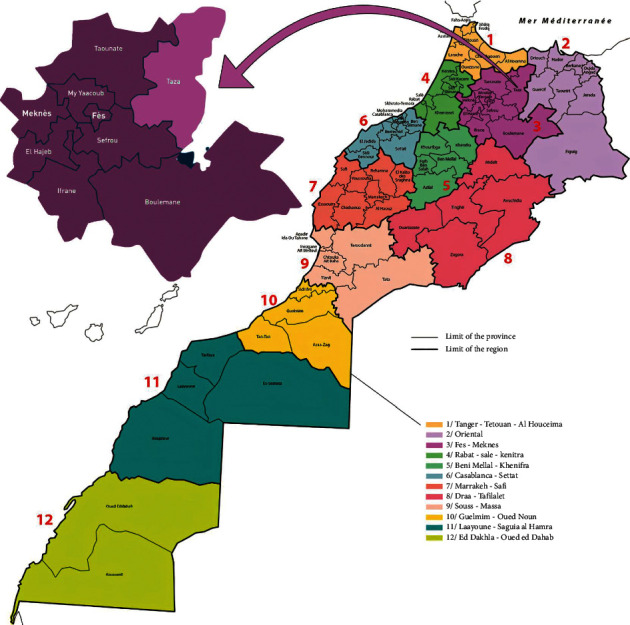
Map of the studied area (northeastern part of Morocco) (source: institutional website of the High Commission for Planning, Kingdom of Morocco, https://www.hcp.ma/region-fes/index.php?start=44).

Taza city is administratively part of the Region of Fez-Meknes, it is located in the northeast of Morocco, and it is located in a mountain pass where the mountain range of the Rif and that of the Middle Atlas meet (Figure 1). Taza province is bordered to the north by the province of Al Hoceima, to the northeast by the province of Nador, to the east by the province of Taourirt, to the south by that of Boulemane, and to the west by the province of Taounate and that of Sefrou (latitude: 34°13′00″N, longitude: 4°01′00″W, and altitude: 550 m). This city covers an area of 37 km^2^ with a population of 148,456 inhabitants in 2014.

### 2.2. Ethnobotanical Survey

The first interview was conducted with the informants, giving them a brief explanation of the objective of the study and the importance of the information they were going to provide in order to sensitize them to participate in this study. In total, 200 people were interviewed directly between May and August 2016 through ethnobotanical surveys in different localities, cities, towns, villages, and douars in the province of Taza.

During the interviews, structured questionnaires were used for data documentation, 20 questionnaires for each zone, and the selected zones were dispersed between the city of Taza, the villages, and the douars of the Rif Mountains and the Middle Atlas belonging administratively to the province of Taza. Each questionnaire consisted of two parts. The first part concerns demographic information such as sex, age, educational level, source of information, and the profession of the participants, while the second part has informative questions on local names of plant species, mode preparation (decoction, maceration, infusion, etc.), the plant part used (stems, roots, leaves, seeds, aerial part, etc.), the method of administration, and the diseases treated by the plants mentioned by the informant. In each interview, the names of the plants were recorded in Moroccan Arabic when they were mentioned.

The botanical materials of 55 plant species were collected from the informants and kept in special glass frames; they were later identified by Pr. Abdelilah Rahou (Faculty of Sciences, Moulay Ismail University, Meknès). The confirmation was carried out by Dr. Mohamed Reda Kachmar using means of the literature.

These samples of plant materials were given herbarium specimen codes, and the voucher plant samples were kept in the Herbarium of the Botany Department of the Scientific Institute of Rabat, Morocco. The complete floristic list was established after the identification and verification of the samples; the identification process was realized using the following references: Moroccan Medicinal and Aromatic Plants [9], Vascular Flora of Morocco [10], Practical Flora of Morocco [10], and Traditional Moroccan Pharmacopoeia [11]. The taxonomy was confirmed on the basis of data available on the International Plant Names Index website: https://www.ipni.org/.

### 2.3. Ethnopharmacological Parameter Analysis

#### 2.3.1. Relative Frequency of Citation

On the basis of the local therapeutic importance of each plant species, the relative frequency of citation (RFC) was calculated according to the following formula [12]:

(1) $$\begin{array}{c} \text{RFC}=\frac{\text{FC}}{N}, \end{array}$$

where FC is the number of participants who mentioned the use of a plant species and *N* is the total number of participants.

### 2.4. Statistical Analysis

The results obtained were processed and analysed using Excel 2010 software.

## 3. Results

### 3.1. Sociodemographic Characteristics

A total of 200 participants comprising herbalists, competent villagers, traditional healers, and normal people from Taza city, including 114 women (57%) and 86 men (43%), were interviewed. Their average age was 52 years with a minimum of 19 years and a maximum of 85 years. The majority of the informants belonged to the rural area (90%), and 61% were illiterate. The majority of participants received their education about herbal medicine from herbalists (54%), while the rest learned from their older family members or from other people (Table 1).

### 3.2. Medicinal Plants

#### 3.2.1. Medicinal Plants Used by the Informants in the Treatment of Various Diseases

**Figure 2:**
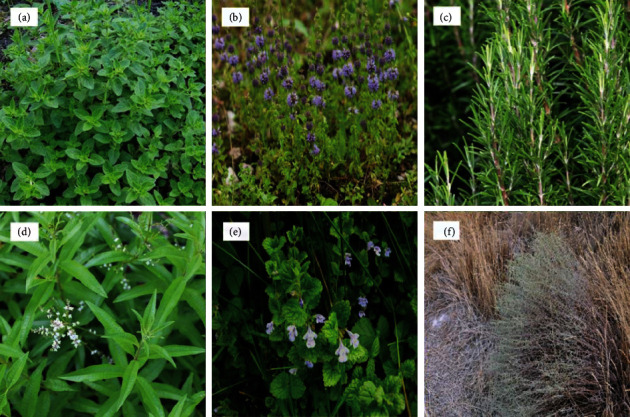
The pictures of (a) *Origanum compactum*, (b) *Mentha pulegium*, (c) *Rosmarinus officinalis L.*, (d) *Aloysia citrodora*, (e) *Calamintha officinalis* Moench, and (f) *Artemisia herba-alba* Asso.

The survey reached 56 plant species used in the treatment of various diseases by the participants in the Taza region. The most used species were *Origanum compactum* with the highest RFC ratio (76%), followed by *Mentha pulegium*, *Rosmarinus officinalis* L., *Aloysia citrodora*, *Calamintha officinalis* Moench, and *Artemisia herba-alba* Asso., with the RFC value of 72%, 60%, 42%, 40%, and 30%, respectively (Table 2). The images of the plants with the highest RFC values are shown in Figure 2.

**Figure 3:**
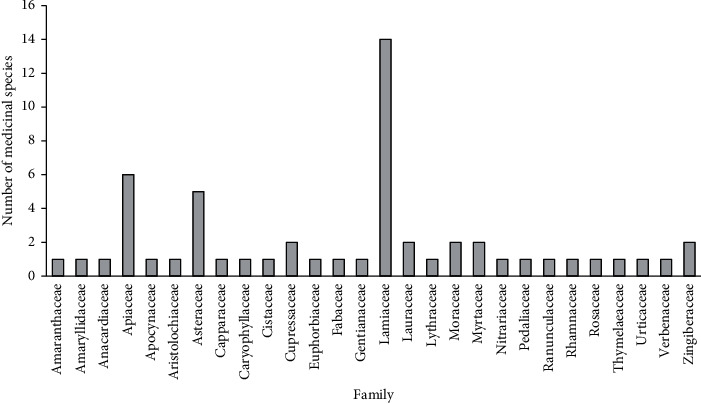
Number of species in each family mentioned by the respondents.

Plants were grouped into 28 families; this census also shows different routes of administration of the drugs, the preparation methods, and the part of the plants used in the traditional medicine as presented in Table 1. The most presented families were Lamiaceae (14 species), Apiaceae (6 species), Asteraceae (5 species), and Myrtaceae (3 species) followed by Cupressaceae, Lauraceae, and Zingiberaceae (2 species). All other families were presented by one species as shown in Figure 3.

### 3.3. Used Parts, Methods of Preparation, and Modes of Administration

**Figure 4:**
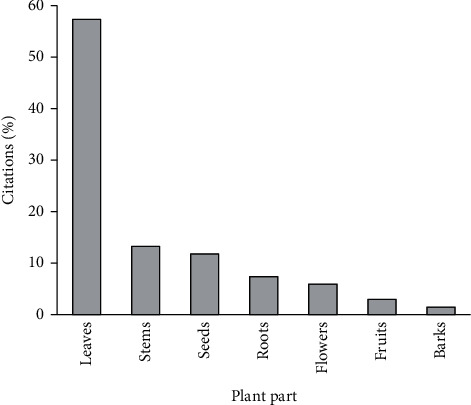
Frequency of different parts used.

**Figure 5:**
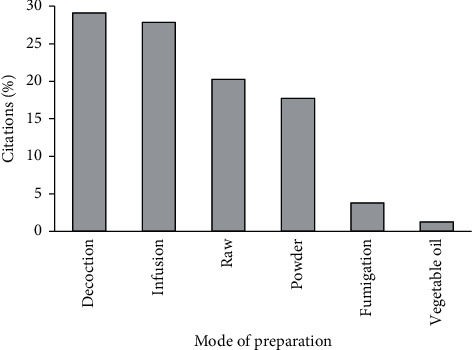
Frequency of different preparation methods.

**Figure 6:**
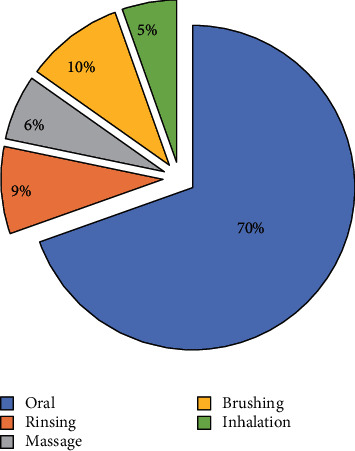
Frequency of the administration mode.

Results obtained in this study showed that leaves were the most used part of the plants (57.35%) followed by the stems (13.23%), seeds (11.76%), roots (7.35%), flowers (5.88%), fruits (2.94%), and barks (1.47%) (Figure 4). Our survey also showed that decoction and infusion were the most used methods of preparation with frequencies of 29.11% and 27.84%, respectively, followed by the raw form (20.25%), powder form (17.72%), fumigation (3.79%), and vegetable oil (1.26%) (Figure 5). Oral administration of the drugs had the highest frequency (70%), while the other administration modes (brushing, rinsing, massage, and inhalation) presented the rest 30% (Figure 6).

## 4. Discussion

The main goal of this study is to identify the medicinal plants used in the province of Taza city. This region has an important and diversified heritage of aromatic and medicinal plants widely used in traditional medicine by the local population. This richness is also reflected by broad culture in phytotherapy and phytopharmacology among the selected informants, particularly herbalists and traditional healers.

Sociodemographic results showed that the age of all participants was between 20 and 86 years, and the most presented group of the informants having the age between 30 and 50 years (48%). The result also showed that females use medicinal plants a little bit more than males. Our results confirm those obtained by other ethnobotanical studies made in other regions in Morocco [7, 128]. This must be due to that women are in charge of drying, stocking of medicinal plants, and preparing recipes for the care of family members.

Interviews showed that older people are particularly competent than the young generation and had a greater knowledge of the uses of medicinal plants for the cure of various diseases; similar results were observed by other studies [30, 129]. However, this finding did not exclude other age groups with valuable knowledge about herbal remedies. In fact, older people are expected to provide more reliable information because they hold more ancestral knowledge transmitted orally. The transmission of this valuable knowledge and medicinal recipes from the old to the new generation is not always assured and is now in decline [129].

In this study, women were the most presented (57%) than men (43%). These results agree with those of a previous ethnobotanical study done in the province of Tata, Souss-Massa region in Morocco [19]. This study was led in the southeast region of Morocco, while our study was conducted in the northeast region of Morocco; these two regions differ by their geographical locations and their climatic zones and consequently a difference in their plant biodiversity, which obviously affects the choice of the plant's species used in traditional medicine. Therefore, our survey showed that the most used plants in the Taza region were *Origanum compactum*, *Mentha pulegium*, *Rosmarinus officinalis*, *Aloysia citrodora*, *Calamintha officinalis* Moench, and *Artemisia herba-alba* Asso., while *Artemisia huguetii*, *Mentha pulegium*, *Trigonella foenum-graecum*, *Mentha suaveolens*, *Lavandula mairei*, and *Nigella sativa* were the most cited for their use in the traditional medicine in the study [19].

Results showed that 61% of the informants were illiterate, and their age was older than 40 years. These results are in agreement with other ethnobotanical studies carried out in Morocco [130] and Algeria [131]. The use of medicinal plants in traditional medicine is more widespread among illiterate people. These results are confirmed by other studies, which have shown that people with a lower level of education have more expertise in the uses of plants in traditional medicine [132, 133]. On the contrary, the results of this survey indicate the predominance of some plant families such as the Lamiaceae, the Apiaceae, and the Asteraceae. The predominance of these families has already been observed in a study carried out in another African country [134] and another study carried out in southeast Morocco [19]. Furthermore, the most used species by the population of Taza province were *Origanum compactum* with the highest RFC (76%), followed by *Mentha pulegium*, *Rosmarinus officinalis*, *Aloysia citrodora Palau*, *Calamintha officinalis* Moench, and *Artemisia herba-alba* Asso., with RFC values of 72%, 60%, 42%, 40%, and 30%, respectively. The medicinal properties that these plants have were experimentally proven by several studies carried out *in vivo* and/or *in vitro* by [135–141]. The plants used mainly by the population of the Taza region are almost the same as those previously mentioned in Morocco [4, 7] and in Algeria [142].

The use of leaves in traditional medicine could be attributed to their availability, the simplicity of their harvest, and their richness in therapeutic substances [143]. On the contrary, decoction was the most used method of preparing medicinal plants (29.11%) followed by infusion (27.84%), the raw form (20.25%), the powder form (17.72%), fumigation (3.79%), and the vegetable oil form (1.26%). This observation is in agreement with other ethnobotanical studies [57], which indicate that the recipes were essentially prepared by decoction, about 67% of herbal preparations were in the liquid form, and water was the solvent of choice in the preparation of herbal recipes because it is abundant and easy to access. The vast majority of remedies were taken orally (70%); similar results have been obtained in other studies [7, 57].

## 5. Conclusion

This study showed that Taza region has a rich and varied patrimony of medicinal plant species used in the folk medicine to treat different diseases. In fact, the traditional recipes based on those plants must be validated and grouped into databases to become as a source for alternative therapeutic compounds, and their use must be conducted by safety and efficacy data, especially for herbalists and traditional healers. Nonetheless, chemical, pharmacological, and toxicological investigations in the medicinal plant area are required to determine and confirm their chemical composition and clinical uses to standardize their correct therapeutic doses.

## Figures and Tables

**Table 1:** Sociodemographic characteristics of the respondents.

Characteristics	Number of informants (*n*)	Frequency (%)
Ages (years)		
18–30	64	32
30–50	96	48
>50	40	20
Total	200	100

Gender		
Male	86	43
Female	114	57
Total	200	100

Education		
Illiterate	122	61
Primary school	26	13
Secondary school	18	9
High school	10	5
University	24	12
Total	200	100

Profession		
Sans	38	19
Peasant	40	20
Housewife	74	37
Herbalist	12	6
Official	30	15
Others	6	3
Total	200	100

Origin of knowledge		
Herbalist	108	54
Popular culture	46	23
Family heritage	32	16
Others	14	7
Total	200	100

**Table 2:** Medicinal plants used in traditional medicine in Taza city region, Morocco.

Family name	Plant species	Voucher codes	Vernacular name	Parts used	Preparation mode	Administration mode	RFC (%)	Utilisation	Ethnomedicinal uses recorded in the literature inside Morocco	Pharmacological properties verified *in vivo* and/or *in vitro*
Amaranthaceae	*Chenopodium ambrosioides* L.	RAB135-16	Mkhinza	Leaves	Raw with juice, raw	Oral Basting	13	Digestive tract infections Asthma Hepatitis	Headaches, migraine, measles, jaundice, syphilis, fever [4]	Antioxidant and immunostimulant [13] Antioxidant, anti-inflammatory, and improvement of intestinal immune status [14]

Amaryllidaceae	*Allium sativum* L.	RAB15-16	Touma	Leaves	Powder	Oral	3	Diabetes Cold Grippe Hypertension	Scorpion and snake bite, intestinal pain, hypertension [1]	Antioxidative and antigenotoxic effects [15]

Anacardiaceae	*Pistacia lentiscus* L.		Drou	Leaves	Decoction	Oral	5	Digestive system pathologies	Digestive diseases and evil eye [1]	Antibacterial activity [16]

Apiaceae	*Daucus crinitus* Desf.		Bozfor	Leaves	Raw Decoction	Oral Inhalation	2	Digestive system disorders	Digestive system [8]	Antimicrobial [17]

Apiaceae	*Foeniculum vulgare* Mill.	RAB92-16	Lbesbas Nafaa	Roots Leaves Seeds	Decoction Infusion Decoction	Oral Oral	4.5	Gastrointestinal diseases Rheumatism Asthma	Mouthwash [18] Kidney diseases, digestive, pain, diabetes [19]	Anti-inflammatory, analgesic, and antioxidant [20] Antibacterial [21]

Apiaceae	*Pimpinella anisum* L.	RAB231-16	Habbat Hlawa	Seeds	Raw	Oral	3	Diabetes, allergy, asthma, digestive system stimulation, tooth care	Spasmolitic, carminative, stomachic, diuretic, expectorant, stimulant kidney diseases, gastric pain, diabetes, antiemetic, tooth care [19]	Antioxidant and antimicrobial [22] Antimicrobial and cytotoxic [23]

Apiaceae	*Petroselinum sativum*	RAB266-16	Maâdnous	Stems Leaves	Decoction	Oral	3	Gastrointestinal infections, heart disease, hypertension, allergy	Cardiac disease and hypertension [24]	Antioxidant and antibacterial [25] Antihypertensive [26]

Apiaceae	*Cuminum cyminum* L.		Lkamoun	Seeds	Infusion	Oral	6	Gastrointestinal infections, stomach ache	Diabetes, cardiovascular diseases, and pathologies of the digestive system [4]	Antimicrobial and cytotoxic activities [27]

Apiaceae	*Coriandrum sativum* L.		Lkazber	Leaves Stems	Infusion	Oral	5.5	Hypertension	Cardiac disease and hypertension [24]	Antioxidant effect [28] Protection of gastric mucosal damage [29]

Apocynaceae	*Nerium oleander* L.	RAB188-16	Defla	Leaves	Infusion Raw	Oral Basting	2	Heart disease, hypertension, diabetes, dermatosis, fever, headache, sciatic nerve pain	Hypertension, cardiac disease, and diabetes [24] Rheumatism, osteoporosis, arthrosis [30]	Antioxidant, antimicrobial, and antitumor [31]

Aristolochiaceae	*Aristolochia longa*		Baraztam	Roots	Decoction Powder	Oral Basting Rinsing	17	Cold, tooth pain, osteoarticular pain, inflammation, allergy	Cardiovascular diseases, neurological diseases [32]	Cytotoxic and antimicrobial [33] Antibacterial [34]

Asteraceae	*Dittrichia viscosa*		Bagramane	Leaves	Raw Infusion	Basting Oral Massage	5	Cold, osteoarticular pain Diabetes Wormer	Dental abscesses [35]	Gastroprotection [36] Antibacterial and antifungal [37] Antibacterial and antioxidant [38]

Asteraceae	*Atractylis gummifera* L.		Addade	Roots	Decoction	Oral	3	Cold Rheumatism Abdominal pain	Tooth whitening, toothache, mouth ulcers, gingival bleeding, gingivitis, herpes labialis, bad breath, stomatitis [35]	Antidiabetic [39]

Asteraceae	*Artemisia herba-alba* Asso.	RAB26-16	Chih	Leaves Stems	Decoction	Oral	30	Gastrointestinal infections Abdominal pain Cold Nausea	Wounds, rheumatism, appetite stimulant, indigestion, diarrhea, bad breath, anthelmintic, emmenagogue, nausea, stomach pain [40]	Nephroprotective [41] Antimicrobial and antioxidant [42] Antioxidant, anticancer, and anti-inflammatory [43]

Asteraceae	*Artemisia absinthium* L.	RAB33-16	Chiba	Leaves	Infusion	Oral	4	Intestinal parasites Dyspepsia Renal colic	Cold and flu, cholagogue, diuretic [44]	Antioxidant and antimicrobial activities [45]

Asteraceae	*Matricaria chamomilla*	RAB151-15	Babounj	Flowers	Infusion Powder	Oral Rinsing	16	Eczema Psoriasis Depression Intestinal colic	Colic, Diarrhée, Nervosité, Depression, Angines, Aphtes, Menstruations douloureuses, Fièvre, Abcès, infections [46]	Antibiofilm and anticaries [47], radical scavenging and antioxidant activity [48]

Capparaceae	*Capparis spinosa* L.		Lkabbar	Fruits	Raw Powder	Oral Basting	6	Digestive tract disorders Dermatological affections Diabetes Helminthiasis Respiratory problems, rheumatic pain, kidney stones	Stomach pain, asthma [40]	Antidiabetic and antihyperlipidemic [49] Antioxidant [50]

Caryophyllaceae	*Herniaria cinerea* DC.		Harast lahjer	Leaves Stems	Decoction	Oral	13	Diabetes Kidney stones	Antiurolithiasis [51] Bladder disorders, kidney stones, diuretic, reduced blood levels of uric acid [52]	Diuretic and decreased renal stone formation [53]

Cistaceae	*Cistus ladanifer* L.	RAB 108848	Touzala lbayda	Leaves	Decoction	Oral	1	Gastric pain	Gastric pain, common cold, and against digestive disorders [54]	Hypoglycemic and hypolipidemic [55] Antibacterial [56]

Cupressaceae	*Juniperus phoenicea*	RAB 108845	Al'Araâr Elbeldi	Leaves	Decoction, raw	Oral	2	Gastrointestinal infections Asthma	Asthma, hepatitis, and rheumatism [57]	Antibacterial activity [58]

Cupressaceae	*Tetraclinis articulata* (Vahl) Mast.	RAB187-16	Al'Araâr	Leaves	Infusion Fumigation	Oral Inhalation	14	Stomach ache Hypotensive Diabetes	Endocrinological, general health, gastrointestinal, otolaryngological, and respiratory [59]	Antioxidant, antimicrobial, anti-inflammatory, and cytotoxic [60] Antibacterial [61]

Euphorbiaceae	*Ricinus communis*		Lkherwaa	Seeds	Oil	Massage Rinsing	8.5	Skin diseases, hair loss	Hair and face care [19]	Antiviral [62]

Fabaceae	*Trigonella foenum-graecum L.*		Lhelba	Seeds	Raw	Oral	4	Stomach ache Diuretic Diabetes	Diabetes, cardiovascular diseases, power problems [4]	Antimicrobial [63] Hypocholesterolemic and anti-inflammatory [64] Antiallergic [65]

Gentianaceae	*Centaurium erythraea* Rafn.	RAB 108847	Gossat lhaya	Leaves Stems	Decoction Powder	Oral Rinsing	9	Diabetes Wound healing Stomach ache Wound inflammation Analgesic	Hepatitis, asthma, and rheumatism [57] Digestive system and kidney diseases [1] Allergy and increasing energy [4]	Antioxidant and anti-inflammatory effects [66] Antihyperglycemic activity [67] Diuretic effects [68]

Lamiaceae	*Mentha pulegium*		Flio	Leaves	Infusion Decoction	Oral	72	Flu Cold	Cold, respiratory canals [1] Pathologies of the digestive system, cold problems, and pathologies of the respiratory system [4]	Antioxidant and antimutagenic activities [69]

Lamiaceae	*Origanum compactum* Benth.		Zaâtar	Leaves	Decoction Infusion Powder	Oral Rinsing	76	Gastrointestinal infection Stomach ache Fever Cold	Emmenagogue, nausea, food poisoning, asthma [40]	Antioxidant and antibacterial activities [70] Antiproliferative effect [71] Antimutagenic effect [72]

Lamiaceae	*Calamintha officinalis* Moench	RAB69-16	Manta	Leaves	Decoction Infusion	Oral	40	Flu Cold	Against different aches, antipyretic [73]	Antioxidant [74] Antioxidant and antimicrobial [75]

Lamiaceae	*Marrubium vulgare* L.	RAB364-16	Mriwta	Leaves	Decoction Raw	Oral Basting	11	Liver disease, respiratory problems, fever, diabetes	Toothache, gingival bleeding, bad breath, gingivitis [35]	Hepatoprotective [76] Antioxidant and antifungal [77]

Lamiaceae	*Mentha rotundifolia*		Mchichetru	Leaves	Infusion	Oral	16	Cold Grippe	Skin pathologies, respiratory disorders, digestive disorders [78]	Anti-inflammatory, analgesic, and antioxidant [79] Insecticidal and antifungal [80]

Lamiaceae	*Ajuga iva* (L.) Schreb.	RAB23-16	Chendgoura	Leaves Stems	Raw Infusion Powder	Oral	4	Diabetes Rheumatism Allergy Digestive disorders Antidiarrhea	Rheumatism, allergy, cancer [57] Cardiovascular diseases, pathologies of the digestive system, pathologies of the respiratory system [4]	Antibacterial activity [81] Antihyperglycemic activity [82,83]

Lamiaceae	*Rosmarinus officinalis* L.		Azir	Leaves Flowers	Decoction Infusion	Oral Massage	60	Gastric disorders Digestive system pathologies Heart disease	Cardiac disease, hypertension, and diabetes [24] Allergy, asthma, cancer, infections, and immune system depression [57] Pathologies of the digestive system, allergy, and dermocosmetology [4]	Antibacterial activity [84] Diuretic effects [68]

Lamiaceae	*Salvia officinalis* L.	RAB354-16	Salmia	Leaves	Infusion	Oral	21	Diabetes	Diabetes [6] Respiratory, digestive, circulatory [85] Cold, cough, diabetes, rheumatism, stomachic, carminative, choleretic, tonic, antisudorific, spasmolytic, throat pain, stomach pain, antiseptic, haemostatic [19]	Antioxidant, antibacterial, and antileishmanial activities [86]

Lamiaceae	*Lavandula stoechas* L.		Lhalhal	Leaves	Infusion	Oral	2.5	Gastrointestinal disorders	Rheumatism and asthma [57] Rheumatism and digestive system [1] Pathologies of the digestive system and diabetes [4]	Antibacterial activity [81]

Lamiaceae	*Thymus vulgaris* L.		Zaitra	Stems Leaves	Decoction	Oral	17	Cold Asthma Digestive tract infections	Colic, Diarrhea, Digestive disorders, Flatulence, Cooling, Bronchitis, Flu, Cough, Toothache, Painful menstruation, and Anemia infections [46] Gum disease, halitosis, oral ulcers [18]	Antioxidant and antibacterial [87] Antioxidative [88]

Lamiaceae	*Lavandula*		Lkhzama	Leaves Flowers	Powder	Oral	20	Urinary system disorder	ND	ND

Lamiaceae	*Mentha spicata* L.		Naanaa	Leaves Stems	Infusion	Oral	4	Cold Grippe	Migraine [89] Respiratory, skin [85]	Antibacterial [90] Antioxidant [91]

Lamiaceae	*Origanum majorana* L.		Mardedoch	Leaves	Infusion Decoction	Oral	7	Gastrointestinal infections Stomach ache Cold	Cephalalgia [40] Gum disease, dental pain [18]	Antidepressant-like effects [92] Antioxidant, antimicrobial, cytotoxicity, and antiacetylcholinesterase [93]

Lamiaceae	*Ocimum basilicum* L.		Hbeq	Leaves	Infusion	Oral	10	Urinary system disorder	Allergy, cardiovascular diseases, and pathologies of the urinary system [4] Against mosquito, sinusitis, and tachycardia [94]	Antibacterial activity of essential oil [84]

Lauraceae	*Laurus nobilis* L.		Warkat sidna mossa	Leaves	Infusion	Oral	7	Respiratory problems Cough Digestive problems	Liver, pancreas, and digestive pain, face care, rheumatism, antiseptic, diuretic, sedative, rheumatism, calefacient [19]	Gastroprotective [95] Antibacterial and antibiofilm [96]

Lauraceae	*Cinnamomum verum* J. Presl		Lkarfa	Bark	Powder	Oral Rinsing	4	Cold Digestive system disorders Diabetes	Emmenagogue, hypercholesterolemia, obesity, painful periods [40]	Antibacterial [97] Antifungal [98]

Lythraceae	*Lawsonia inermis* L.		Lhana	Leaves	Powder	Basting	8	Hair protection Wound healing	Hair care, antifungal, burns, sprains, hypotensive, emetic, stomach pains, digestive disorders [19]	Antibacterial and antifungal [99] Wound healing [100] Antibacterial [101]

Moraceae	*Ficus carica* L.	RAB82-16	Chriha Lkarmous	Fruits	Raw	Oral	1.5	Asthma	Digestive system [1] Pathologies of the digestive system, pathologies of the circulatory system, and cardiovascular diseases [4]	Anticancer [102] Hepatoprotective and nephroprotective [103]

Myrtaceae	*Eucalyptus globulus* Labill (sp)	RAB93-16	Al'Kalitouss	Leaves	Fumigation	Inhalation	11	Flu	Diabetes [6] Asthma [57]	Antibacterial activity [104] Hypoglycemic activity [105]

Myrtaceae	*Myrtus communis* L.	RAB496-16	Arraihan	Leaves	Raw Decoction	Massage Oral	13	Hair loss Diarrhea	Diabetes [6] Cardiac weakness, digestive system [1]	Antioxidant activity [106] Antigenotoxic effect [107] Hypoglycemic effect [108]

Myrtaceae	*Eugenia caryophyllata*	RAB412-16	Qronfel	Flowers	Decoction Powder	Inhalation Massage Rinsing	3	Grippe Tooth pain	Headaches, migraine, pathologies of the digestive system, dermocosmetology [4]	Antibacterial and antioxidant [109] Antioxidant capacity and cytotoxic activity [110]

Nitrariaceae	*Peganum harmala* L.		Lharmal	Seeds	Fumigation	Inhalation	6.5	Rheumatism, back pain, fever	Gingivitis, toothache, mouth ulcers, herpes labialis, bad breath, stomatitis [35] Spasmolitic, sterility, uterus diseases, vermifuge, abortifacient, ritual, magic practice, and to relieve bad fate, hair care, eczema, neoplasms [19]	Antiviral [111] Antibacterial and antifungal [112]

Pedaliaceae	*Sesamum indicum* L.	RAB528-16	Ajenjlane	Seeds	Raw	Oral	1	Digestive system disorders	Appetite stimulant [40] Bloating, digestion problems [73]	Antiulcer [113] Gastroprotective [114] Antirheumatoid [115]

Ranunculaceae	*Nigella sativa* L.	RAB358-16	Lhaba sawda Sanûj	Seeds	Oil	Massage	2	Eczema Psoriasis	Appetite stimulant, kidney diseases, cough [40]	Nephron-protective [116] Antibacterial [117]

Rhamnaceae	*Ziziphus lotus* (L.) Lam.	RAB622-16	Sedra	Leaves Stems	Infusion	Oral	1.5	Headache Joint pain	Cardiac ailments, pulmonary infection, haemostatic, colic animals, diabetes, stomach pain, diarrhea, kidney stones, throat pain, pectoral and emollient, jaundice [19]	Antiglycaemic, anticholesterolemic, antioxidant, and antimicrobial [118]

Rosaceae	*Alchemilla vulgaris*		Gdam sbaâ	Leaves	Raw Powder	Rinsing	4.5	Wound healing Inflammation of wounds	ND	ND

Thymelaeaceae	*Daphne gnidium* L.		Lzaz	Leaves	Raw	Basting	9	Hair loss	Hair care and hair strengthening [1] Dermocosmetology, fever, and head problems [4]	Anti-inflammatory [119] Antimicrobial [120]

Urticaceae	*Urtica dioica* L.	RAB565-16	Lhriga	Leaves	Decoction	Oral	8	Urinary system problem	Diabetes [6] Osteoporosis [30] Renal weakness, digestive system [1]	Antibacterial [121] Antioxidant, antimicrobial, antiulcer, and analgesic [22]

Verbenaceae	*Aloysia citrodora* Palau		Lwiza	Leaves	Infusion Decoction	Oral	42	Stomach ache Hypertension Diabetes	Sedative, hypertension, cold [40] Digestive, antiseptic, carminative, sedative, gastric lavage, calming, calefacient [19]	Antioxidant activity and antimicrobial properties [122]

Zingiberaceae	*Curcuma longa* L.		Lkharkoum	Roots	Powder	Basting	1	Pain Skin diseases	Dermatological, genitourinary, hepatic [32] Urinary-genital, mouth, breast, lung, digestive [123]	Antibacterial [124] Cytotoxic, antioxidant, and anti-inflammatory [125]

Zingiberaceae	*Zingiber officinale* Roscoe.		Skine jbir	Roots	Cooked powder	Oral	3	Cough	Aphrodisiac, cold, asthma, bronchitis, calefacient, depurative, analgesic, spice, digestive [19]	Antioxidant activity [126] Antibacterial [127]

## Data Availability

The data used to support the findings of this study are included within the article.

## References

[1] El-hilaly J., Hmammouchi M., Lyoussi B. (2003). Ethnobotanical studies and economic evaluation of medicinal plants in Taounate province (Northern Morocco). *Journal of Ethnopharmacology*.

[2] Barkaoui M., Katiri A., Boubaker H., Msanda F. (2017). Ethnobotanical survey of medicinal plants used in the traditional treatment of diabetes in Chtouka Ait Baha and Tiznit (Western Anti-Atlas), Morocco. *Journal of Ethnopharmacology*.

[3] Ullah M., Khan M. U., Mahmood A. (2013). An ethnobotanical survey of indigenous medicinal plants in Wana district south Waziristan agency, Pakistan. *Journal of Ethnopharmacology*.

[4] Fakchich J., Elachouri M. (2014). Ethnobotanical survey of medicinal plants used by people in Oriental Morocco to manage various ailments. *Journal of Ethnopharmacology*.

[5] Holaly G. E., Simplice K. D., Charlemagne G. (2015). Étude ethnobotanique des plantes utilisées dans le traitement du diabète dans la médecine traditionnelle de la région Maritime du Togo. *Pan African Medical Journal*.

[6] Mrabti H. N., Jaradat N., Kachmar M. R. (2019). Integrative herbal treatments of diabetes in *Beni Mellal* region of Morocco. *Journal of Integrative Medicine*.

[7] Bouyahya A., Abrini J., Et-touys A., Bakri Y., Dakka N. (2017). Indigenous knowledge of the use of medicinal plants in the North-West of Morocco and their biological activities. *European Journal of Integrative Medicine*.

[8] El Haouari M., El Makaoui S., Jnah M., Haddaouy A. (2018). A survey of medicinal plants used by herbalists in Taza (Northern Morocco) to manage various ailments. *Journal of Materials and Environmental Science*.

[9] Hmamouchi M. (1999). Les plantes médicinales et aromatiques marocaines : Utilisation, biologie, écologie, chimie, pharmacologie, toxicologie, lexiques, IDPCM, mohammedia.

[10] Fennane M., Ibn Tattou M., Mathez J. (1999). Practical flora of Morocco: Manual for the determination of vascular plants. *Botanical Series*.

[11] Bellakhdar J. (1997). La pharmacopée marocaine traditionnelle, 2ème édition augmen. https://lefennec.com/livre/la-pharmacopee-marocaine-traditionnelle-jamal-bellakhdar/.

[12] Yetein M. H., Houessou L. G., Lougbégnon T. O., Teka O., Tente B. (2013). Ethnobotanical study of medicinal plants used for the treatment of malaria in plateau of Allada, Benin (West Africa). *Journal of Ethnopharmacology*.

[13] Maldonado-Garcia M., Angulo C., Vazquez-Martinez J., Sanchez V., Lopez M. G., Reyes-Becerril M. (2019). Antioxidant and immunostimulant potentials of *Chenopodium ambrosioides* L. in Pacific red snapper (*Lutjanus peru*). *Aquaculture*.

[14] Reyes-Becerril M., Angulo C., Sanchez V., Vázquez-Martínez J., López M. G. (2019). Antioxidant, intestinal immune status and anti-inflammatory potential of *Chenopodium ambrosioides* L. in fish: In vitro and in vivo studies. *Fish & Shellfish Immunology*.

[15] Nencini C., Menchiari A., Franchi G. G., Micheli L. (2011). In vitro antioxidant activity of aged extracts of some Italian Allium species. *Plant Foods for Human Nutrition*.

[16] Derwich E., Manar A., Benziane Z., Boukir A. (2010). GC/MS analysis and in vitro antibacterial activity of the essential oil isolated from leaf of *Pistacia lentiscus* growing in Morocoo. *World Applied Sciences Journal*.

[17] Dib M. A., Bendahou M., Bendiabdellah A. (2010). Partial chemical composition and antimicrobial activity of *Daucus crinitus Desf*. extracts. *Grasas y Aceites*.

[18] Zougagh S., Belghiti A., Rochd T. (2019). Medicinal and aromatic plants used in traditional treatment of the oral pathology: the ethnobotanical survey in the economic capital casablanca, Morocco (North Africa). *Natural Products and Bioprospecting*.

[19] Abouri M., El Mousadik A., Msanda F., Boubake H., Saadi B., Cherifi K. (2012). An ethnobotanical survey of medicinal plants used in Rwanda for voluntary depigmentation. *International Journal of Medicinal Plants Research*.

[20] Choi E.-M., Hwang J.-K. (2004). Antiinflammatory, analgesic and antioxidant activities of the fruit of *Foeniculum vulgare*. *Fitoterapia*.

[21] Lo Cantore P., Iacobellis N. S., De Marco A., Capasso F., Senatore F. (2004). Antibacterial activity of *Coriandrum sativum* L. and *Foeniculum vulgare Miller var*. *vulgare* (miller) essential oils. *Journal of Agricultural and Food Chemistry*.

[22] Gülçin İ., Küfrevioǵlu Ö. İ., Oktay M., Büyükokuroǵlu M. E. (2004). Antioxidant, antimicrobial, antiulcer and analgesic activities of nettle (*Urtica dioica* L.). *Journal of Ethnopharmacology*.

[23] Abdel-Reheem M. A. T., Oraby M. M. (2015). Anti-microbial, cytotoxicity, and necrotic ripostes of *Pimpinella anisum* essential oil. *Annals of Agricultural Sciences*.

[24] Eddouks M., Maghrani M., Lemhadri A., Ouahidi M.-L., Jouad H. (2002). Ethnopharmacological survey of medicinal plants used for the treatment of diabetes mellitus, hypertension and cardiac diseases in the south-east region of Morocco (Tafilalet). *Journal of Ethnopharmacology*.

[25] Wong P., Kitts D. (2006). Studies on the dual antioxidant and antibacterial properties of parsley (*Petroselinum crispum*) and cilantro (*Coriandrum sativum*) extracts. *Food Chemistry*.

[26] Ajebli M., Eddouks M. (2019). Antihypertensive activity of *Petroselinum crispum* through inhibition of vascular calcium channels in rats. *Journal of Ethnopharmacology*.

[27] Abbaszadegan A., Gholami A., Ghahramani Y. (2016). Antimicrobial and cytotoxic activity of *Cuminum cyminum* as an intracanal medicament compared to chlorhexidine gel. *Iranian Endodontic Journal*.

[28] Deepa B., Anuradha C. V. (2011). Antioxidant potential of *Coriandrum sativum* L. seed extract. *Indian Journal of Experimental Biology*.

[29] Al-Mofleh I. A., Alhaider A. A., Mossa J. S., Al-Sohaibani M. O., Rafatullah S., Qureshi S. (2006). Protection of gastric mucosal damage by *Coriandrum sativum* L. pretreatment in *Wistar albino* rats. *Environmental Toxicology and Pharmacology*.

[30] Chaachouay N., Benkhnigue O., Fadli M., El Ayadi R., Zidane L. (2019). Ethnobotanical study of medicinal plants used to treat osteoarticular diseases in the Moroccan Rif, Morocco. *Journal of Pharmacy and Pharmacognosy Research*.

[31] Suganya R. S., Priya K., Roxy B. S. (2012). Phytochemical screening and antibacterial activity from nerium oleander and evaluvate their plant mediated nanoparticle synthesis. *International Research Journal of Pharmacy*.

[32] Ben Akka F., Salhi S., Benkhnigue O., Dahmani J., Douira A., Zidane L. (2019). Ethnobotanical study of medicinal plants used in the region of middle oum Rbia (Morocco). *Plant Archives*.

[33] Hinou J., Demetzos C., Harvala C., Roussakis C. (1990). Cytotoxic and antimicrobial principles from the roots of *Aristolochia longa*. *International Journal of Crude Drug Research*.

[34] El Omari N., Akkaoui S., El Blidi O. (2020). HPLC-DAD/TOF-MS chemical compounds analysis and evaluation of antibacterial activity of *Aristolochia longa* root extracts. *Natural Product Communications*.

[35] Najem M., Harouak H., Ibijbijen J., Nassiri L. (2020). Oral disorders and ethnobotanical treatments: a field study in the central Middle Atlas (Morocco). *Heliyon*.

[36] Alarcon De La Lastra C., Lopez A., Motilva V. (1993). Gastroprotection and prostaglandin E2 generation in rats by flavonoids of *Dittrichia viscosa*. *Planta Medica*.

[37] Rhimi W., Ben Salem I., Immediato D., Saidi M., Boulila A., Cafarchia C. (2017). Chemical composition, antibacterial and antifungal activities of crude *Dittrichia viscosa* (L.) greuter leaf extracts. *Molecules (Basel, Switzerland)*.

[38] Gharred N., Dbeibia A., Falconieri D., Hammami S., Piras A., Dridi-Dhaouadi S. (2019). Chemical composition, antibacterial and antioxidant activities of essential oils from flowers, leaves and aerial parts of *Tunisian Dittrichia Viscosa*. *Journal of Essential Oil Research*.

[39] Bouabid K., Lamchouri F., Toufik H., Sayah K., Cherrah Y., Faouzi M. E. A. (2018). Phytochemical screening and in vitro evaluation of alpha amylase, alpha glucosidase and beta galactosidase inhibition by aqueous and organic *Atractylis gummifera* L. extracts. *Plant Science Today*.

[40] Idm’Hand E., Msanda F., Cherifi K. (2020). Ethnobotanical study and biodiversity of medicinal plants used in the Tarfaya province, Morocco. *Shengtai Xuebao/Acta Ecologica Sinica*.

[41] Sekiou O., Boumendjel M., Taibi F., Tichati L., Boumendjel A., Messarah M. (2021). Nephroprotective effect of *Artemisia herba alba* aqueous extract in alloxan-induced diabetic rats. *Journal of Traditional and Complementary Medicine*.

[42] Mighri H., Hajlaoui H., Akrout A., Najjaa H., Neffati M. (2010). Antimicrobial and antioxidant activities of Artemisia herba-alba essential oil cultivated in Tunisian arid zone. *Comptes Rendus Chimie*.

[43] Khlifi D., Sghaier R. M., Amouri S., Laouini D., Hamdi M., Bouajila J. (2013). Composition and anti-oxidant, anti-cancer and anti-inflammatory activities of *Artemisia herba-alba*, *Ruta chalpensis* L. and *Peganum harmala* L. *Food and Chemical Toxicology*.

[44] Jaadan H., Akodad M., Moumen A. (2020). Ethnobotanical survey of medicinal plants growing in the region of “Oulad daoud zkhanine” (Nador province), in Northeastern Morocco. *Ethnobotany Research and Applications*.

[45] Karabegović I., Nikolova M., Veličković D., Stojičević S., Veljković V., Lazić M. (2011). Comparison of antioxidant and antimicrobial activities of methanolic extracts of the *Artemisia* sp. recovered by different extraction techniques. *Chinese Journal of Chemical Engineering*.

[46] Mikou K., Rachiq S., Jarrar Oulidi A. (2016). Étude ethnobotanique des plantes médicinales et aromatiques utilisées dans la ville de Fès au Maroc. *Phytothérapie*.

[47] Braga A. S., de M. Simas L. L., Pires J. G. (2020). Antibiofilm and anti-caries effects of an experimental mouth rinse containing *Matricaria chamomilla* L. extract under microcosm biofilm on enamel. *Journal of Dentistry*.

[48] Kolodziejczyk-Czepas J., Bijak M., Saluk J. (2015). Radical scavenging and antioxidant effects of *Matricaria chamomilla* polyphenolic-polysaccharide conjugates. *International Journal of Biological Macromolecules*.

[49] Mollica A., Zengin G., Locatelli M. (2017). Anti-diabetic and anti-hyperlipidemic properties of *Capparis spinosa* L.: In vivo and in vitro evaluation of its nutraceutical potential. *Journal of Functional Foods*.

[50] Jiménez-López J., Ruiz-Medina A., Ortega-Barrales P., Llorent-Martínez E. J. (2018). Phytochemical profile and antioxidant activity of caper berries (*Capparis spinosa* L.): evaluation of the influence of the fermentation process. *Food Chemistry*.

[51] Boufous H., Marhoume F., Chait A., Bagri A. (2017). Ethnopharmacological survey of medicinal plants with hallucinogenic effect and plants used against pain, inflammatory diseases, diabetes and urinary lithiasis in Zagora “Morocco”. *Journal of Intercultural Ethnopharmacology*.

[52] El Midaoui M., Maataoui A., Benbella M., Ait Houssa A., Labazi N. (2011). Ethnobotanical study of some aromatic and medicinal plants in the Middle Atlas Mountains of Morocco. *Natural Product Communications*.

[53] Atmani F., Slimani Y., Mimouni M., Aziz M., Hacht B., Ziyyat A. (2004). Effect of aqueous extract from *Herniaria hirsuta* L. on experimentally nephrolithiasic rats. *Journal of Ethnopharmacology*.

[54] Merzouki A., Ed-derfoufi F., Molero Mesa J. (2000). Contribution to the knowledge of Rifian traditional medicine II: Folk medicine in Ksar Lakbir district (NW Morocco). *Fitoterapia*.

[55] El Kabbaoui M., Chda A., Azdad O. (2016). Evaluation of hypoglycemic and hypolipidemic activities of aqueous extract of *Cistus ladaniferus* in streptozotocin-induced diabetic rats. *Asian Pacific Journal of Tropical Biomedicine*.

[56] Benali T., Bouyahya A., Habbadi K. (2020). Chemical composition and antibacterial activity of the essential oil and extracts of *Cistus ladaniferus* subsp. ladanifer and *Mentha suaveolens* against phytopathogenic bacteria and their ecofriendly management of phytopathogenic bacteria. *Biocatalysis and Agricultural Biotechnology*.

[57] Youbi A., Ouahidi I., Mansouri L., Daoudi A., Bousta D. (2016). Ethnopharmacological survey of plants used for immunological diseases in four regions of Morocco. *European Journal of Medicinal Plants*.

[58] Derwich E., Benziane Z., Boukir A. (2010). Chemical composition of leaf essential oil of juniperus phoenicea and evaluation of its antibacterial activity. *International Journal of Agriculture and Biology*.

[59] Teixidor-Toneu I., Martin G. J., Ouhammou A., Puri R. K., Hawkins J. A. (2016). An ethnomedicinal survey of a Tashelhit-speaking community in the High Atlas, Morocco. *Journal of Ethnopharmacology*.

[60] Rached W., Zeghada F. Z., Bennaceur M. (2018). Phytochemical analysis and assessment of antioxidant, antimicrobial, anti-inflammatory and cytotoxic properties of *Tetraclinis articulata* (Vahl) Masters leaves. *Industrial Crops and Products*.

[61] Achmit M., Aoussar N., Mellouki F. (2021). In vitro antibacterial and biofilm inhibitory activity of the sawdust essential oil of *Tetraclinis articulata* (vahl) against catheter-associated *Staphylococcus aureus* clinical isolates. *Current Research in Biotechnology*.

[62] Elkousy R. H., Said Z. N. A., Abd El-Baseer M. A., Abu El wafa S. A. (2021). Antiviral activity of castor oil plant (*Ricinus communis*) leaf extracts. *Journal of Ethnopharmacology*.

[63] Subhapriya S., Gomathipriya P. (2018). Green synthesis of titanium dioxide (TiO_2_) nanoparticles by *Trigonella foenum-graecum* extract and its antimicrobial properties. *Microbial Pathogenesis*.

[64] Cheurfa M., Allem R., Sadeer N. B., Mahomoodally M. F. (2021). In vivo hypocholesterolemic and anti-inflammatory effect of *Aloysia triphylla* (L’Hér.) Britton and *Trigonella foenum-græcum* L. seeds. *South African Journal of Botany*.

[65] Bae M.-J., Shin H. S., Choi D.-W., Shon D.-H. (2012). Antiallergic effect of *Trigonella foenum-graecum* L. extracts on allergic skin inflammation induced by trimellitic anhydride in BALB/c mice. *Journal of Ethnopharmacology*.

[66] Kachmar M. R., Oliveira A. P., Valentão P. (2019). HPLC-DAD-ESI/MS^n^ phenolic profile and in vitro biological potential of *Centaurium erythraea* Rafn aqueous extract. *Food Chem.*.

[67] Mansar-benhamza L., Djerrou Z., Hamdi Pacha Y. (2013). Evaluation of anti-hyperglycemic activity and side effects of *Erythraea centaurium* (L.) Pers. in rats. *African Journal of Biotechnology*.

[68] Haloui M., Louedec L., Michel J.-B., Lyoussi B. (2000). Experimental diuretic effects of *Rosmarinus officinalis* and *Centaurium erythraea*. *Journal of Ethnopharmacology*.

[69] Yumrutas O., Saygideger S. D. (2012). Determination of antioxidant and antimutagenic activities of *Phlomis armeniaca* and *Mentha pulegium*. *Journal of Applied Pharmaceutical Science*.

[70] Bouhdid S., Skali S. N., Idaomar M. (2008). Antibacterial and antioxidant activities of *Origanum compactum* essential oil. *African Journal of Biotechnology*.

[71] Chaouki W., Leger D. Y., Eljastimi J., Beneytout J.-L., Hmamouchi M. (2010). Antiproliferative effect of extracts from *Aristolochia baetica* and *Origanum compactumon* human breast cancer cell line MCF-7. *Pharmaceutical Biology*.

[72] Mezzoug N., Elhadri A., Dallouh A. (2007). Investigation of the mutagenic and antimutagenic effects of *Origanum compactum* essential oil and some of its constituents. *Mutation Research/Genetic Toxicology and Environmental Mutagenesis*.

[73] Mechachate H., Es-safi I., Jawhari F., Bari A., Grafov A., Bousta D. (2020). Ethnobotanical survey about the management of diabetes with medicinal plants used by diabetic patients in Region of Fez-Meknès Morocco. *Ethnobotany Research and Applications*.

[74] Hayani M., Benhlima N., Bouzoubaa A. (2020). Phytochemical study, polyphenols determination and evaluation of antioxidant activity of *Origanum compactum* and *Satureja calamintha nepeta* from the region of Ouazzane (Morocco). *Mediterranean Journal of Chemistry*.

[75] Cherrat L., Espina L., Bakkali M., Pagán R., Laglaoui A. (2014). Chemical composition, antioxidant and antimicrobial properties of *Mentha pulegium*, *Lavandula stoechas* and *Satureja calamintha* Scheele essential oils and an evaluation of their bactericidal effect in combined processes. *Innovative Food Science & Emerging Technologies*.

[76] Akther N., Shawl A. S., Sultana S., Chandan B. K., Akhter M. (2013). Hepatoprotective activity of *Marrubium vulgare* against paracetamol induced toxicity. *Journal of Pharmacy Research*.

[77] Rezgui M., Majdoub N., Mabrouk B. (2020). Antioxidant and antifungal activities of marrubiin, extracts and essential oil from *Marrubium vulgare* L. against pathogenic dermatophyte strains. *Journal de Mycologie Médicale*.

[78] El Hassani F. Z. (2020). Characterization, activities, and ethnobotanical uses of Mentha species in Morocco. *Heliyon*.

[79] Boussouf L., Boutennoune H., Kebieche M., Adjeroud N., Al-Qaoud K., Madani K. (2017). Anti-inflammatory, analgesic and antioxidant effects of phenolic compound from *Algerian Mentha rotundifolia* L. leaves on experimental animals. *South African Journal of Botany*.

[80] Yakhlef G., Hambaba L., Pinto D. C. G. A., Silva A. M. S. (2020). Chemical composition and insecticidal, repellent and antifungal activities of essential oil of *Mentha rotundifolia* (L.) from Algeria. *Industrial Crops and Products*.

[81] Bouyahya A., Abrini J., El-baabou A., Bakri Y., Dakka N. (2016). Determination of phenol content and antibacterial activity of five medicinal plants ethanolic extracts from North-west of Morocco. *Journal of Plant Pathology and Microbiology*.

[82] Hilaly J. E., Lyoussi B. (2002). Hypoglycaemic effect of the lyophilised aqueous extract of *Ajuga iva* in normal and streptozotocin diabetic rats. *Journal of Ethnopharmacology*.

[83] El-Hilaly J., Tahraoui A., Israili Z. H., Lyoussi B. (2007). Acute hypoglycemic, hypocholesterolemic and hypotriglyceridemic effects of continuous intravenous infusion of a lyophilised aqueous extract of *Ajuga iva* L. Schreber whole plant in streptozotocin-induced diabetic rats. *Pakistan Journal of Pharmaceutical Sciences*.

[84] Talbaoui A. (2012). Chemical composition and antibacterial activity of essential oils from six Moroccan plants. *Journal of Medicinal Plant Research*.

[85] Ouhaddou H., Boubaker H., Msanda F., El Mousadik A. (2015). An ethnobotanical study of medicinal plants of the Agadir Ida Ou Tanane province (Southwest Morocco). *Journal of Applied Biosciences*.

[86] Et-Touys A., Fellah H., Mniouil M. (2016). Screening of antioxidant, antibacterial and antileishmanial activities of *Salvia officinalis* L. Extracts from Morocco. *British Microbiology Research Journal*.

[87] Nadia Z., Rachid M. (2013). Antioxidant and antibacterial activities of thymus vulgaris. *Medicinal and Aromatic Plants Research Journals*.

[88] Chizzola R., Michitsch H., Franz C. (2008). Antioxidative properties of Thymus vulgaris leaves: Comparison of different extracts and essential oil chemotypes. *Journal of Agricultural and Food Chemistry*.

[89] Chaachouay N., Benkhnigue O., Zidane L. (2020). Ethnobotanical study aimed at investigating the use of medicinal plants to treat nervous System diseases in the Rif of Morocco. *Journal of Chiropractic Medicine*.

[90] Boukhebti H., Chaker A. N., Belhadj H. (2011). Chemical composition and antibacterial activity of *Mentha pulegium* L. and *Mentha spicata* L. essential oils. *Der Pharmacia Lettre*.

[91] Kanatt S. R., Chander R., Sharma A. (2007). Antioxidant potential of mint (*Mentha spicata* L.) in radiation-processed lamb meat. *Food Chemistry*.

[92] Abbasi-Maleki S., Kadkhoda Z., Taghizad-Farid R. (2020). The antidepressant-like effects of *Origanum majorana* essential oil on mice through monoaminergic modulation using the forced swimming test. *Journal of Traditional and Complementary Medicine*.

[93] Hajlaoui H., Mighri H., Aouni M., Gharsallah N., Kadri A. (2016). Chemical composition and in vitro evaluation of antioxidant, antimicrobial, cytotoxicity and anti-acetylcholinesterase properties of *Tunisian Origanum majorana* L. essential oil. *Microbial Pathogenesis*.

[94] Bellakhdar J., Claisse R., Fleurentin J., Younos C. (1991). Repertory of standard herbal drugs in the *Moroccan pharmacopoea*. *Journal of Ethnopharmacology*.

[95] Afifi F. U., Khalil E., Tamimi S. O., Disi A. (1997). Evaluation of the gastroprotective effect of *Laurus nobilis* seeds on ethanol induced gastric ulcer in rats. *Journal of Ethnopharmacology*.

[96] Merghni A., Marzouki H., Hentati H., Aouni M., Mastouri M. (2016). Antibacterial and antibiofilm activities of *Laurus nobilis* L. essential oil against *Staphylococcus aureus* strains associated with oral infections. *Current Research in Translational Medicine*.

[97] Choi O., Cho S. K., Kim J., Park C. G., Kim J. (2016). In vitro antibacterial activity and major bioactive components of *Cinnamomum verum* essential oils against cariogenic bacteria, *Streptococcus mutans* and *Streptococcus sobrinus*. *Asian Pacific Journal of Tropical Biomedicine*.

[98] Mariappan P. M., Sabesan G., Koilpillai B., Janakiraman S., Sharma N. K. (2013). Chemical characterisation and antifungal activity of methanolic extract of *Cinnamomum verum* J. Presl bark against Malassezia spp. *Pharmacognosy Journal*.

[99] Yusuf M., Ahmad A., Shahid M. (2012). Assessment of colorimetric, antibacterial and antifungal properties of woollen yarn dyed with the extract of the leaves of henna (*Lawsonia inermis*). *Journal of Cleaner Production*.

[100] Jridi M., Sellimi S., Lassoued K. B. (2017). Wound healing activity of cuttlefish gelatin gels and films enriched by henna (*Lawsonia inermis*) extract. *Colloids and Surfaces A: Physicochemical and Engineering Aspects*.

[101] Jeyaseelan E. C., Jenothiny S., Pathmanathan M., Jeyadevan J. (2012). Antibacterial activity of sequentially extracted organic solvent extracts of fruits, flowers and leaves of *Lawsonia inermis* L. from Jaffna. *Asian Pacific Journal of Tropical Biomedicine*.

[102] Mousa F., Ali A., Nasir M., Khattak K. (2021). Comparative anticancer activities of *Ficus carica* and *Ficus salicifolia* latex in MDA-MB-231 cells. *Saudi Journal of Biological Sciences*.

[103] Fouad D., Alhatem H., Abdel-Gaber R., Ataya F. (2019). Hepatotoxicity and renal toxicity induced by gamma-radiation and the modulatory protective effect of *Ficus carica* in male albino rats. *Research in Veterinary Science*.

[104] Bachir R. G., Benali M. (2012). Antibacterial activity of the essential oils from the leaves of Eucalyptus globulus against *Escherichia coli* and *Staphylococcus aureus*. *Asian Pacific Journal of Tropical Biomedicine*.

[105] Jouad H., Lemhadri A., Maghrani M., Burcelin R., Eddouks M. (2003). Hawthorn evokes a potent anti-hyperglycemic capacity in streptozotocin-induced diabetic rats. *Journal of Herbal Pharmacotherapy*.

[106] Amensour M., Sendra E., Abrini J., Bouhdid S., Pérez-Alvarez J. A., Fernández-López J. (2009). Total phenolic content and antioxidant activity of myrtle (*Myrtus communis*) extracts. *Natural Product Communications*.

[107] Hayder N., Abdelwahed A., Kilani S. (2004). Anti-genotoxic and free-radical scavenging activities of extracts from (Tunisian) *Myrtus communis*. *Mutation Research/Genetic Toxicology and Environmental Mutagenesis*.

[108] Sepici A., Gürbüz I., Çevik C., Yesilada E. (2004). Hypoglycaemic effects of myrtle oil in normal and alloxan-diabetic rabbits. *Journal of Ethnopharmacology*.

[109] El amrani S., El Ouali Lalami A., Ez zoubi Y., Moukhafi K., Bouslamti R., Lairini S. (2019). Evaluation of antibacterial and antioxidant effects of cinnamon and clove essential oils from Madagascar. *Materials Today: Proceedings*.

[110] Figueiredo P. L. B., Pinto L. C., da Costa J. S. (2019). Composition, antioxidant capacity and cytotoxic activity of *Eugenia uniflora* L. chemotype-oils from the Amazon. *Journal of Ethnopharmacology*.

[111] Moradi M.-T., Karimi A., Rafieian-Kopaei M., Fotouhi F. (2017). In vitro antiviral effects of Peganum harmala seed extract and its total alkaloids against Influenza virus. *Microbial Pathogenesis*.

[112] Nenaah G. (2010). Antibacterial and antifungal activities of (beta)-carboline alkaloids of *Peganum harmala* (L) seeds and their combination effects. *Fitoterapia*.

[113] Hazarika I., Hussain M., Das A. (2015). Anti-Ulcer activity of ethanolic extract of *Sesamum indicum* seed on indomethacin-induced ulcer model and its antioxidant property in Gastro-protection. *Research & Reviews: A Journal of Pharmacology*.

[114] Okwuosa C. N., Okoi-Ewa R., Achukwu P. U., Onuba A. C., Azubuike N. C. (2011). Gastro-protective effect of crude hexane leaf extract of *Sesamum indicum* in rabbits. *Nigerian Journal of Physiological Sciences*.

[115] Ruckmani A., Meti V., Vijayashree R. (2018). Anti-rheumatoid activity of ethanolic extract of *Sesamum indicum* seed extract in Freund’s complete adjuvant induced arthritis in Wistar albino rats. *Journal of Traditional and Complementary Medicine*.

[116] Farooqui Z., Ahmed F., Rizwan S., Shahid F., Khan A. A., Khan F. (2017). Protective effect of Nigella sativa oil on cisplatin induced nephrotoxicity and oxidative damage in rat kidney. *Biomedicine & Pharmacotherapy*.

[117] Rathi G., Siddiqui S. I., Pham Q., Nam V. T. (2020). Nigella sativa seeds based antibacterial composites: a sustainable technology for water cleansing-a review. *Sustainable Chemistry and Pharmacy*.

[118] Dahlia F., Barouagui S., Hemida H., Bousaadia D., Rahmoune B. (2020). Influence of environment variations on anti-glycaemic, anti-cholesterolemic, antioxidant and antimicrobial activities of natural wild fruits of *Ziziphus lotus* (L.). *South African Journal of Botany*.

[119] Harizi H., Chaabane F., Ghedira K., Chekir-Ghedira L. (2011). Inhibition of proinflammatory macrophage responses and lymphocyte proliferation in vitro by ethyl acetate leaf extract from *Daphne gnidium*. *Cellular Immunology*.

[120] Cottigli F., Loy G., Garau D. (2001). Antimicrobial evaluation of coumarins and flavonoids from the stems of L. *Phytomedicine*.

[121] Salih N. A. (2014). Antibacterial effect of nettle (*Urtica dioica*). *Al-Qadisiyah Journal of Veterinary Medicine Sciences*.

[122] Hashemi S. M. B., Mousavi Khaneghah A., Koubaa M. (2018). Extraction of essential oil from *Aloysia citriodora Palau* leaves using continuous and pulsed ultrasound: Kinetics, antioxidant activity and antimicrobial properties. *Process Biochemistry*.

[123] Alami Merrouni I., Elachouri M. (2021). Anticancer medicinal plants used by Moroccan people: ethnobotanical, preclinical, phytochemical and clinical evidence. *Journal of Ethnopharmacology*.

[124] Naz S., Jabeen S., Ilyas S., Manzoor F., Aslam F., Ali A. (2010). Antibacterial activity of Curcuma longa varieties against different strains of bacteria. *Pakistan Journal of Botany*.

[125] Ramsewak R. S., DeWitt D. L., Nair M. G. (2000). Cytotoxicity, antioxidant and anti-inflammatory activities of curcumins I-III from *Curcuma longa*. *Phytomedicine*.

[126] Stoilova I., Krastanov A., Stoyanova A., Denev P., Gargova S. (2007). Antioxidant activity of a ginger extract (*Zingiber officinale*). *Food Chemistry*.

[127] Malu S., Obochi G., Tawo E., Nyong B. (2009). Antibacterial activity and medicinal properties of ginger (*Zingiber officinale*). *Global Journal of Pure and Applied Sciences*.

[128] Chaachouay N., Benkhnigue O., Fadli M., El Ibaoui H., Zidane L. (2019). Ethnobotanical and ethnopharmacological studies of medicinal and aromatic plants used in the treatment of metabolic diseases in the Moroccan Rif. *Heliyon*.

[129] Anyinam C. (1995). Ecology and ethnomedicine: exploring links between current environmental crisis and indigenous medical practices. *Social Science & Medicine*.

[130] Daoudi A., Bammou M., Zarkani S., Slimani I., Ibijbijen J., Nassiri L. (2015). Étude ethnobotanique de la flore médicinale dans la commune rurale d’Aguelmouss province de Khénifra (Maroc). *Phytothérapie*.

[131] Miara M. D., Bendif H., Rebbas K., Rabah B., Hammou M. A., Maggi F. (2019). Medicinal plants and their traditional uses in the highland region of Bordj Bou Arreridj (Northeast Algeria). *Journal of Herbal Medicine*.

[132] Wassie S. M., Aragie L. L., Taye B. W., Mekonnen L. B. (2015). Knowledge, attitude, and utilization of traditional medicine among the communities of Merawi town, northwest Ethiopia: a cross-Sectional study. *Evidence-Based Complementary and Alternative Medicine*.

[133] Chaachouay N., Benkhnigue O., Fadli M., El Ibaoui H., El Ayadi R., Zidane L. (2019). Ethnobotanical and ethnopharmacological study of medicinal and aromatic plants used in the treatment of respiratory System disorders in the Moroccan Rif. *Ethnobotany Research and Applications*.

[134] El-mokasabi F. M., Al-sanousi M. F., El-mabrouk R. M. (2018). Taxonomy and ethnobotany of medicinal plants in eastern region of Libya. *Journal of Environmental Science, Toxicology and Food Technology*.

[135] Chahbi A., Nassik S., El Amri H. (2020). Chemical composition and antimicrobial activity of the essential oils of two aromatic plants cultivated in Morocco (*Cinnamomum cassia* and *Origanum compactum*). *Journal of Chemistry*.

[136] Wani A. R., Yadav K., Khursheed A., Rather M. A. (2020). An updated and comprehensive review of the antiviral potential of essential oils and their chemical constituents with special focus on their mechanism of action against various influenza and coronaviruses. *Microbial Pathogenesis*.

[137] Karadağ A. E., Demirci B., Çaşkurlu A. (2019). In vitro antibacterial, antioxidant, anti-inflammatory and analgesic evaluation of *Rosmarinus officinalis* L. flower extract fractions. *South African Journal of Botany*.

[138] Murino Rafacho B. P., Dos Santos P. P., Gonçalves A. D. F. (2017). Rosemary supplementation (*Rosmarinus oficinallis* L.) attenuates cardiac remodeling after myocardial infarction in rats. *PLoS One*.

[139] Oukerrou M. A., Tilaoui M., Mouse H. A., Leouifoudi I., Jaafari A., Zyad A. (2017). Chemical composition and cytotoxic and antibacterial activities of the essential oil of *Aloysia citriodora* Palau Grown in Morocco. *Advances in Pharmacological Sciences*.

[140] Bertella A., Benlahcen K., Abouamama S. (2018). *Artemisia herba-alba Asso*. essential oil antibacterial activity and acute toxicity. *Industrial Crops and Products*.

[141] Gharzouli K., Khennouf S., Amira S., Gharzouli A. (1999). Effects of aqueous extracts from *Quercus ilex* l. root bark, *Punica granatum* l. fruit peel and *Artemisia herba-alba Asso* leaves on ethanol-induced gastric damage in rats. *Phytotherapy Research*.

[142] Miara M. D., Bendif H., Ait Hammou M., Teixidor-Toneu I. (2018). Ethnobotanical survey of medicinal plants used by nomadic peoples in the Algerian steppe. *Journal of Ethnopharmacology*.

[143] Tra Bi F., Irie G., N’Gaman K., Mahou C. (2008). Études de quelques plantes thérapeutiques utilisées dans le traitement de l\’hypertension artérielle et du diabète : Deux maladies émergentes en Côte d\’Ivoire. *Science of Nature*.

